# Geospatial analysis of area-level social and behavioral factors associated with short interpregnancy intervals in North Carolina, U.S.

**DOI:** 10.1016/j.pmedr.2025.103216

**Published:** 2025-08-22

**Authors:** Qian Huang, Liane M. Ventura, Jo Bradley, Kate Beatty

**Affiliations:** a Department of Biostatistics and Epidemiology, College of Public Health, East Tennessee State University, Box 70259, Johnson City, TN 37614-1700, United States of America; b Center for Applied Research and Evaluation in Women's Health, College of Public Health, East Tennessee State University, Box 70264, Johnson City, TN 37614-1700, United States of America; c Department of Epidemiology and Community Health, University of North Carolina at Charlotte, 9201 University City Blvd, Charlotte, NC 28223, United States of America; d WNC Health Network, 1 Haywood Street, Suite 425, Asheville, NC 28801, United States of America

**Keywords:** Pregnancy planning, Short interpregnancy intervals, Social factors, Health behaviors, Spatial lag model

## Abstract

**Objective:**

Short interpregnancy intervals (sIPI), defined as six months or less, are associated with adverse birth outcomes and are influenced by socioeconomic factors, which reflect a complex interplay between health outcomes, social factors, health behaviors, and geographic contexts. This study aims to examine the spatial distribution of sIPI across North Carolina, in the Southeast United States (U.S.), and assess its association with social and behavioral factors.

**Methods:**

We used secondary data from the North Carolina State Center for Health Statistics and the U.S. Census Bureau spanning 2018–2022 to analyze the spatial distribution of sIPI. Associations between sIPI and social and behavioral factors—including race, fertility age, educational attainment, poverty rates, transportation, prenatal smoking, prenatal care in the first trimester, and breastfeeding initiation rates—were assessed using ordinary least squares and spatial lag models.

**Results:**

County-level analyses revealed significant spatial autocorrelation of sIPI in North Carolina. Mecklenburg County had the highest number of cases (*n* = 6255), while Jones County had the highest percentage (18.9 %). No significant urban–rural differences were found. High-high clusters were primarily located in western Mountain and southern Coastal counties. SIPI was positively associated with the percentage of reproductive-age females and limited vehicle access, with a significant spatial lag term indicating spatial dependence.

**Conclusions:**

Findings emphasize the need for targeted public health interventions such as comprehensive school-based sexual health education and group-level prenatal care programs to reduce the prevalence of sIPI, prevent subsequent adverse birth outcomes, and mitigate geographic health disparities in North Carolina, U.S.

## Highlights

• Short interpregnancy interval varies geographically across North Carolina.
• Clusters found in the mountain and coastal regions of North Carolina.
• No urban–rural difference in short interpregnancy interval rates.
• Lack of vehicles and more women of reproductive age are linked to short intervals.
• Findings support area-level interventions and local public health programs.

## 1 Introduction

In the United States (U.S.), approximately 30 % of interpregnancy intervals – the time between last birth and start of a subsequent pregnancy - were shorter than eighteen months between 2015 and 2022, with 5 % occurring within less than six months (Admon et al., 2025). Short interpregnancy intervals (sIPI), of six months or less, are associated with elevated risks of adverse birth outcomes, including preterm and spontaneous preterm birth (Class et al., 2017; Hanley et al., 2017; Tessema et al., 2021). However, no nationally established medical practice guidelines exist regarding optimal pregnancy spacing (Ahrens et al., 2019). The relationship between sIPI and adverse birth outcomes in high-resource settings is attenuated by confounding social and behavioral factors (Ahrens et al., 2018). Understanding social and behavioral factors impacting sIPI, particularly on an area-level within geographic context, is important to inform interventions and policy.

SIPI may increase the risk of adverse birth outcomes among predisposed individuals with lower socioeconomic status (Appareddy et al., 2017; Kaharuza et al., 2001). Additionally, lack of transportation or access to prenatal care, not planning to breastfeed, and smoking during pregnancy could increase rates of sIPI (McFarland et al., 2023). Low levels of social capital and unintended pregnancy are also associated with sIPI (Gemmill and Lindberg, 2013). In Australia, Yang et al. (2019) found that, among women with an interpregnancy interval less than twelve months, 52.3 % desired longer birth spacing. Components of social capital include domestic and neighborhood cohesion, social networks, and participation in and contribution to ones' community (Agampodi et al., 2019). County-level social capital is positively correlated with adverse birth outcomes, suggesting a complex or even detrimental effect in certain contexts (Shoff and Yang, 2013). At the individual-level, decision-making is strongly influenced by subjective norms including others' behaviors within a peer group (DeMaria et al., 2019). Given the complexities of social and behavioral factors associated with sIPI, particularly within a geographic context, a thorough area-level examination is warranted.

Previous studies have investigated the geographic distribution of breastfeeding rates in relation to programmatic interventions and social factors (Grubesic and Durbin, 2016; Liberty et al., 2019). Research from Ethiopia demonstrated significant spatial autocorrelation of sIPI, with geographic clusters of elevated risk influenced by individual- and community-level factors (Kassie et al., 2023). In the U.S., spatial nonstationarity has been observed in various health outcomes, highlighting the varying influence of contextual variables on health across regions (Huang, 2023, Huang, 2024; Kwan, 2021). However, few studies have examined maternal health outcomes in a geographically diverse state like North Carolina, within the Southeast U.S., which encompasses three distinct regions – Mountain, Piedmont, and Coastal – each with its own cultural, socioeconomic, and racial compositions. The Mountain region, in particular, has faced the closure of labor and delivery units, which have disproportionally impacted vulnerable women, particularly those covered by Medicaid (Sullivan et al., 2021).

This study fills the gap by examining how the distribution of sIPI aligns with social factors (e.g., race, age, educational attainment, poverty, and household vehicle access) and perinatal health behaviors (e.g., prenatal smoking, prenatal care in the first trimester, and breastfeeding initiation rates) across North Carolina from 2018 to 2022. First, we analyze the geospatial distribution of sIPI across the state and the variability between urban and rural geographies. Then we explore how sIPI relates to social factors and perinatal health behaviors. Findings from this study aim to advance the understanding of how social and behavioral factors are related to area-level rates of sIPI and the role of geography within these relationships. The study seeks to inform public health programs and health policy to reduce sIPI and associated adverse perinatal outcomes in North Carolina and other areas throughout the U.S.

## 2 Methods

### 2.1 Data sources

North Carolina operates a decentralized public health system where each county provides services. County-level data were used, as counties represent functional and policy-relevant administrative units for health planning and intervention in North Carolina and the U.S. (Stoto et al., 2019). Data sources include (1) North Carolina State Center for Health Statistics, covering the percentage of sIPI, prenatal care, smoking during pregnancy, and breastfeeding at discharge from 2018 to 2022, (2) demographic and socioeconomic indicators from the U.S. Census Bureau, including maternal age, race, educational attainment, poverty status, and household vehicle access from 2018 to 2022 and (3) Rural-Urban Continuum Codes (RUCC) from the 2023 U.S. Department of Agriculture Economic Research Service.

### 2.2 Measures

**Fig. 1:**
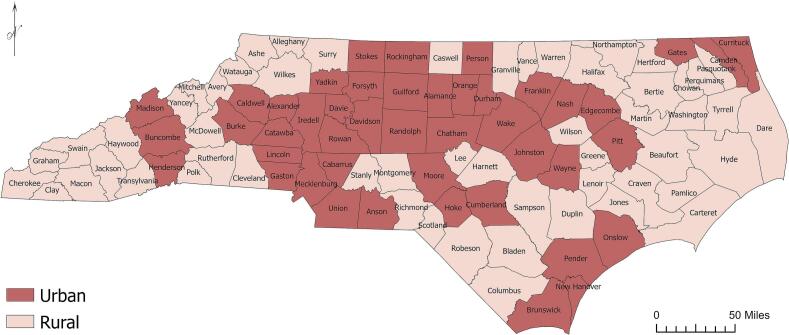
Urban and rural county designations across North Carolina based on United States Department of Agriculture Economic Research Service 2023 classifications.

SIPI is defined as births where the interval between the previous birth and conception of a subsequent pregnancy is six months or less, including both hospital and home births. Prenatal care is measured by the percentage of mothers who initiated care during the first trimester, a widely used indicator of adequate prenatal care (Bloch et al., 2009). The RUCC codes are grouped into urban (codes one through three) and rural categories (codes four through nine). Of North Carolina's one hundred counties, forty-five were urban and fifty-five rural (Fig. 1). Details on all variables and sources are provided in Table 1. All data used are publicly available.

**Table 1:** Description, sources, and rationale for variables used in the analysis of short interpregnancy interval among women in North Carolina, 2018–2022.

Variable	Timeframe	Variable Description	Source	Rationale
Short interpregnancy interval	2018–2022	Percent short interpregnancy intervals - Interval from last birth to conception of next pregnancy is six months or less	North Carolina State Center for Health Statistics	Ahrens et al., 2019
Race	2018–2022	Percent of White	U.S. Census Bureau	Lonhart et al., 2019
Maternal age	2018–2022	Female population age 14–44	U.S. Census Bureau	Gemmill and Lindberg, 2013; Shoff and Yang, 2013; Thoma et al., 2016
Education	2018–2022	Percent of Bachelor's degrees or higher for population 18 years and older	U.S. Census Bureau	Thoma et al., 2016
Poverty	2018–2022	Percent of population 18 years and older below federal poverty level	U.S. Census Bureau	Appareddy et al., 2017; McFarland et al., 2023
Household vehicle access	2018–2022	Percent of occupied housing units with no household vehicles available	U.S. Census Bureau	Appareddy et al., 2017; McFarland et al., 2023
Smoking during pregnancy	2018–2022	Percent of mothers who smoked during pregnancy	North Carolina State Center for Health Statistics	Appareddy et al., 2017; McFarland et al., 2023
Prenatal care	2018–2022	Percent of mothers who received prenatal care in the 1st trimester	North Carolina State Center for Health Statistics	Appareddy et al., 2017; McFarland et al., 2023
Breastfeeding at discharge	2018–2022	Percent of live infants who initiated breastfeeding between delivery and discharge from birthing facility	North Carolina State Center for Health Statistics	McFarland et al., 2023
Urban/rural	2023	Rural-urban Continuum Codes [RUCC]	USDA Economic Research Service	Lemas et al., 2023; Shoff and Yang, 2013

Abbreviations: U.S. – United States; USDA – United States Department of Agriculture.

### 2.3 Statistical analysis and models

The percentage of sIPI was analyzed using Global and Optimized Local Moran's I to evaluate spatial autocorrelation. Global Moran's I provided an overall measure of spatial autocorrelation for the entire state, while Local Moran's I identified county-level clusters and outliers. An inverse distance spatial relationship was applied, with Euclidean distance as the metric. Urban-rural differences in sIPI were compared using independent samples *t*-tests, with significance set at the 0.05 level.

To examine the county-level sIPI in relation to social and behavioral variables, sIPI rates served as the outcome variable. Eight explanatory variables include: race (percent of White population), fertility age (females aged 15–44), educational attainment (bachelor's degree or higher), poverty rates, transportation (no household vehicle access), prenatal smoking, prenatal care in the first trimester, and breastfeeding initiation at hospital discharge. All predictors were expressed as percentages and retained in their original scale. *Z*-score standardization was not applied to preserve coefficient interpretability in terms of percentage-point changes. Bivariate associations between sIPI and predictors were tested using Spearman's rho (two-tailed, alpha = 0.05).

An ordinary least squares (OLS) regression was used as the baseline model. We assessed heteroskedasticity using the Breusch-Pagan and Koenker-Bassett tests. Spatial dependence in OLS residuals was evaluated using Global Moran's I and the Lagrange Multiplier test to determine the need for spatial modeling. A spatial lag model was then fitted to account for neighboring counties' influence and improve coefficient precision. We used a first-order rook contiguity spatial weights matrix, which defined counties as neighbors only if they shared a boundary (edge). This approach was chosen over queen contiguity (which includes corners) to more conservatively define neighborhood structure and reduce potential over-specification (Mawarni and Machdi, 2016). County neighbors were identified using Federal Information Processing Standards codes, and the matrix was row-standardized. On average, counties had approximately five neighbors, capturing the underlying spatial structure of the study area. All analyses were conducted using ArcGIS Pro 3.0.3 (Esri, Redlands, California), GeoDa 1.22.0.10, and IBM SPSS Statistics 28.0 (IBM Corp., Armonk, New York).

This study was exempt from Institutional Review Board review under the guidelines of East Tennessee State University. Analyses were based on publicly available aggregated data and did not involve identifiable information.

## 3 Results

### 3.1 Spatial distribution of sIPI in North Carolina

**Fig. 2:**
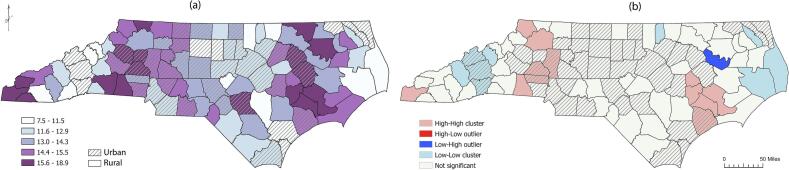
County-level short interpregnancy interval percentages and spatial clustering across North Carolina, 2018–2022. *Note: Panel (a) shows the county-level percentage of short interpregnancy intervals. Panel (b) shows Local Moran's I cluster categories and definitions: high–high (counties with high values surrounded by high values; light red), low–low (counties with low values surrounded by low values; light blue), high–low outliers (counties with high values surrounded by low values; bright red), and low–high outliers (counties with low values surrounded by high values; bright blue).* (For interpretation of the references to colour in this figure legend, the reader is referred to the web version of this article.)

In North Carolina, there were 53,933 cases of sIPI between 2018 and 2022, representing 13.3 % of the 405,511 second or higher-order births. This percentage excludes first births from the total of 596,495 births, as IPI does not apply to first pregnancies. The highest number of sIPI occurred in Mecklenburg County, with 6255 cases, accounting for 12.7 % of total births (*n* = 73,803). However, the highest percentage of sIPI was found in Jones County, with 55 cases, representing 18.9 % of total births (*n* = 454). The lowest numbers of sIPI were recorded in Hyde County, with 10 cases, representing 7.7 % of total births (*n* = 178), and Dare County, with 74 cases, representing 7.5 % of total births (*n* = 1546) (Fig. 2a).

Global Moran's I statistic indicated a statistically significant clustering of sIPI across the state (Moran's *I* = 0.53, z-score = 7.24, *p* < 0.01). Local Moran's I analysis (Fig. 2b) revealed significant clusters and outliers. High-high clusters (areas of high sIPI surrounded by high sIPI) were observed in the western mountain region (Cherokee County). High-high clusters were also found in Ashe, Wilkes, Iredell, Catawba, Lincoln, and Cleveland Counties, as well as in coastal counties including Greene, Lenoir, Craven, Jones, and Onslow Counties. Low-low clusters (areas of low sIPI surrounded by low sIPI) were found in the mid-western mountain region, including Madison, Haywood, Buncombe, Henderson, and Yancey Counties, and the northeast coastal region, such as Vance, Pasquotank, Hyde, and Dare Counties. There was one low-high outlier (an area with low sIPI values surrounded by high sIPI values), located in Martin County, where 104 cases accounted for 13.0 % of total births.

### 3.2 Rural and urban differences

The *t*-test results showed no significant difference in the rates of sIPI between rural and urban counties in North Carolina between 2018 and 2022 (t (98) = −0.37, *p* = 0.72). The mean percentage of sIPI in rural areas was 13.56 % (SD = 2.54), while in urban areas it was slightly lower at 13.38 % (SD = 2.25). However, the difference was not statistically significant, suggesting the variance of sIPI was similar in rural and urban locations.

### 3.3 Underlying social and behavioral factors of sIPI

The analyses revealed a moderate and significant negative correlation between sIPI and the percentage of individuals with a bachelor's degree or higher and breastfeeding initiation, *r* = −0.30 and − 0.42 (*p* < 0.01), respectively. A moderate and significant positive correlation was found between sIPI and poverty rates (*r* = 0.26, p < 0.01), no household vehicle access (*r* = 0.30, p < 0.01), and prenatal smoking (0.25, *p* < 0.05) (Table 2).

**Table 2:** Spearman correlation coefficients between short interpregnancy interval and county-level social and behavioral factors in North Carolina (100 counties), 2018–2022.

	Short Interpregnancy Interval	Race	Maternal Age	Education	Poverty	Household Vehicle Access	Smoking during Pregnancy	Prenatal Care	Breastfeeding at Discharge
Short Interpregnancy Interval	–	−0.05	−0.01	−0.30**	0.26**	0.30**	0.25*	−0.05	−0.42**
Race	−0.05	–	−0.31**	0.30**	−0.46**	−0.55**	0.43**	0.44**	0.46**
Maternal Age	−0.01	−0.31**	–	0.23*	−0.07	0.04	−0.48**	−0.23*	0.18
Education	−0.30**	0.30**	0.23*	–	−0.61**	−0.42**	−0.43**	0.23*	0.74**
Poverty	0.26**	−0.46**	−0.07	−0.61**	–	0.68**	0.28**	−0.23*	−0.62**
Household Vehicle Access	0.30**	−0.55**	0.04	−0.42**	0.68**	–	0.20*	−0.15	−0.61**
Smoking during Pregnancy	0.25*	0.43**	−0.48**	−0.43**	0.28**	0.20*	–	0.16	−0.40**
Prenatal Care	−0.05	0.44**	−0.23*	0.23*	−0.23*	−0.15	0.16	–	0.19
Breastfeeding at Discharge	−0.42**	0.46**	0.18	0.74**	−0.62**	−0.61**	−0.40**	0.19	–

Statistical test: Spearman's rho (2-tailed): * p < 0.05; ** *p* < 0.01 (2-tailed).

Variable definitions and measurement scale (All variables are continuous percentages at the county level; none were analyzed as categorical or dichotomous in this table):

Short interpregnancy interval: Percent of births where the interval from previous birth to conception is six months or less.

Race: Percent of residents who are White.

Reproductive-age females (15–44 years): Percent of the population who are females aged fifteen to forty-four years.

Educational attainment (bachelor's degree or higher): Percent of adults aged eighteen years and older with a bachelor's degree or higher.

Poverty: Percent of adults aged eighteen years and older below the federal poverty level.

Households without a vehicle: Percent of occupied housing units with no vehicle available.

Prenatal smoking: Percent of mothers who smoked during pregnancy.

Prenatal care in first trimester: Percent of mothers who initiated prenatal care during the first trimester.

Breastfeeding initiation at discharge: Percent of live births where breastfeeding was initiated between birth and hospital discharge.

The county-level percentages of White residents, fertility-age females (aged 15–44), and individuals who received prenatal care in the first trimester were not significantly associated with sIPI. Interestingly, while race (percentage of White residents) showed no significant relationship with sIPI, it was significantly associated with all other explanatory variables, suggesting an underlying racial disparity among social and behavioral factors across the state (Table 2).

Initially, OLS showed significance for sIPI rates (Adjusted R^2^ = 0.18, *p* < 0.01, AICc = 448.64) based on all eight explanatory variables (Appendix 1). Multicollinearity diagnostics showed that all predictors had Variance Inflation Factor (VIF) scores below 5 (range: 1.34–4.89), indicating that multicollinearity was not a major concern (Kim, 2019). In this model, only the percentage of females aged 15–44 was statistically significant (*t* = 2.50, p < 0.01), suggesting that counties with higher percentages of females of childbearing age are more likely to have higher sIPI rates. The percentage of adults with a bachelor's degree or higher (β = −0.08, *p* = 0.06) and households without vehicle access (β = 0.28, p = 0.06) approached statistical significance, suggesting a negative association with educational attainment and a positive association with lack of transportation, respectively.

The OLS model showed low prediction power and positive spatial autocorrelation in its residual (Moran's *I* = 0.28, z = 5.15, *p* < 0.01), indicating spatial dependencies not fully captured by the model. Meanwhile, tests for heteroskedasticity indicated no significant evidence of non-constant variance in the residuals, as both the Breusch–Pagan (χ^2^ = 5.77, *p* = 0.67) and Koenker–Bassett (χ^2^ = 8.71, *p* = 0.37) tests were not statistically significant.

To address spatial dependence, Lagrange Multiplier tests were conducted, supporting the use of a spatial lag model (Lagrange Multiplier =26.86, Robust Lagrange Multiplier =10.98, *p* < 0.01), with a non-significant robust error term (Robust Lagrange Multiplier error = 2.38, *p* = 0.12) (Appendix 2). The spatial lag model demonstrated improved model performance compared to OLS, with an R^2^ of 0.46, log-likelihood of −202.81, and AICc of 425.61. SIPI rates were positively associated with the percentage of females of reproductive age (ρ = 0.11, *p* = 0.01) and households without vehicles (ρ = 0.21, *p* = 0.07). Specifically, higher proportions of women aged 15–44 and households without access to a vehicle were associated with higher county-level sIPI rates. The spatially lagged dependent variable (W_sIPI) was statistically significant and positively associated with sIPI rates (ρ = 0.58, p < 0.01), indicating strong spatial dependence. The Likelihood Ratio Test (χ^2^ = 25.02, p < 0.01) further confirmed that the spatial lag model provided a significantly better fit than the OLS model. Other variables were not significantly associated with sIPI rates in the model.

## 4 Discussion

This study highlights spatial disparities in sIPI across North Carolina, in the Southeast U.S., from 2018 to 2022. A total of 53,933 sIPI cases (13.3 % of all 405,511 births) were recorded across the state. Geographic variations of sIPI were evident, with high-high clusters identified in the Mountain (Cherokee County), Piedmont (Ashe, Wilkes, Iredell, Catawba, Lincoln, and Cleveland Counties), and Coastal regions (Greene, Lenoir, Craven, Jones, and Onslow Counties).

Results revealed significant correlations between sIPI and social and behavioral factors. County-level sIPI rates were significantly lower in areas with higher percentages of individuals holding a bachelor's degree or higher. Similarly, sIPI rates decreased as the proportion of individuals initiating breastfeeding increased. A significant positive correlation was observed between sIPI and poverty rates, lack of household vehicle access, and prenatal smoking — meaning that as poverty, transportation barriers, or prenatal smoking rates increase, so does the prevalence of sIPI. These findings are consistent with previous studies, which found that educational attainment (Thoma et al., 2016) and breastfeeding initiation (Khoshnood et al., 1998) are protective against sIPI, while poverty rates (Appareddy et al., 2017), lack of transportation (McFarland et al., 2023), and prenatal smoking (Khoshnood et al., 1998) are associated with increased risk. Additionally, county-level spatial regression analyses demonstrate significant associations between sIPI and the proportion of females of reproductive age and lack of household vehicle access.

Findings suggest the importance of area-level social capital and individual-level subjective norms on sIPI. Area-level social capital, defined as resources accessible through social networks and community ties, could have adverse effects on health outcomes when negative health behaviors are embedded within a community, including smoking during pregnancy (Shoff and Yang, 2013). At the individual level, subjective norms, reflecting a person's perceptions of the behaviors expected by significant others in their community, have the potential to influence long-acting reversible contraceptive use (DeMaria et al., 2019), duration of breastfeeding (Zhu et al., 2017), and breast self-examination for cancer screening (Norman and Cooper, 2011). Subjective norms regarding ideal pregnancy intervals vary by cultural, social, and economic contexts (Liauw et al., 2019; Taylor et al., 2022). While some women may view closely spaced pregnancies as desirable because of advancing maternal age or career planning, others may favor longer intervals for health or economic considerations (Taylor et al., 2022). However, women with shorter IPI had lower rates of planned pregnancy compared to those with longer intervals (Liauw et al., 2019).

Supporting these points, the percentage of the female population aged 15–44 showed a strong overall correlation with sIPI across North Carolina, even when controlling for other social factors (*t* = 2.50, *p* = 0.01). This variable may capture both community-level social dynamics (e.g., prevailing norms around ideal family size or timing) and social capital-related influences (e.g., peer or familial reinforcement of closely spaced pregnancies). These findings suggest that areas with a higher concentration of women of reproductive age are more susceptible to the influence of local norms and social structures that promote shorter IPI or lack holistic education about family planning and postpartum contraceptive method options.

While no statistically significant differences in sIPI were identified between rural and urban counties, maternity care provider shortages remain a significant challenge in rural North Carolina. Maternity care access designations are based on three factors: the ratio of obstetric clinicians to births, the availability of birthing facilities, and the proportion of women without health insurance (Stoneburner et al., 2024). The distribution of perinatal health care services throughout North Carolina is inconsistent, particularly in rural areas where labor and delivery units have closed, resulting in travel time up to 185 min to a planned birthing facility (Woodward et al., 2023). Jones County, which is a rural county within a high-high sIPI cluster in the Coastal region, had the highest county-level percentage of sIPI (18.9 %, 55 cases out of 454 births). Jones County is currently classified as a maternity care desert, facing both provider shortages and limited access to health care services.

Our findings underscore the importance of access to high-quality obstetrics services that provide patient-centered contraceptive care. The lack of access to transportation is a known barrier to postpartum individuals accessing postpartum follow-up visits, which is an important point of access for postpartum contraception (Cross-Barnet et al., 2018). Conversely, having access to transportation for high-quality family planning services may increase the uptake of postpartum contraception (Karra et al., 2022).

Although prenatal smoking was not statistically significant in the regression models, it showed a meaningful relationship in the correlation analysis. Cherokee County, identified as a high-high sIPI cluster, had a prenatal smoking rate of 19.3 %, notably higher than the statewide average of 6.5 %. The prevalence of smoking during pregnancy is associated with disadvantaged socioeconomic status and social inequalities (Bonello et al., 2023). Additionally, women who reported unwanted pregnancies were more likely to report adverse perinatal health behaviors, including prenatal smoking (Robbins et al., 2021).

While not significant in the spatial models, breastfeeding initiation rates were negatively correlated with sIPI across North Carolina, suggesting that breastfeeding is protective against sIPI. McFarland et al. (2023) suggested that women with sIPI are less likely to intend to breastfeed their infants. However, longer durations of breastfeeding may protect against sIPI (Damtie et al., 2021; de Bocanegra et al., 2014). A possible explanation is that, while an unreliable form of long-term contraception, lactational amenorrhea may protect against immediate postpartum conception due to the release of the prolactin hormone, which inhibits ovulation (Van der Wijden and Manion, 2015).

## 5 Limitations

This study is not without limitations. While U.S. public health policy aims to reduce the proportion of pregnancies conceived within eighteen months of the previous birth (Office of Disease Prevention and Health Promotion, 2022), research suggests that sIPI of less than six months following the previous birth are at the highest risk of adverse birth outcomes (Ahrens et al., 2018). The measure of sIPI in this study is consistent with the highest risk interval of six months or less and provides a targeted assessment of such high-risk birth intervals. While this study assessed a variety of social factors and perinatal health behaviors, additional measures would be important to enhance our understanding of geographic variations in healthcare access on sIPI. Such measures would include the availability of obstetricians/gynecologists, health insurance coverage, and antenatal or birth care services, assessed through multiple dimensions of healthcare access (Penchansky and Thomas, 1981). Additionally, while VIF scores did not indicate problematic multicollinearity, moderate correlations among predictors may have weakened some associations in the multivariable model. Finally, although county-level data are justified by their practical relevance for public health and policy, they may be too coarse to capture localized variations. Future studies using finer spatial units, such as census tracts or block groups, could provide more detailed insights into area-level risk factors, particularly in a geographically diverse state like North Carolina.

## 6 Implications for policy and practice

Findings from this study suggest there is no one-size-fits-all approach to addressing sIPI in North Carolina. While North Carolina's Perinatal Health Strategic Plan (2022–2026) details state-level goals to improve birth outcomes and reduce inequities across the state (Johnson et al., 2022), localized interventions are required based on the unique social needs of each county. Ongoing county-level community health assessments and health improvement plans have the potential to mitigate sIPI by including data related to maternal health and birth outcomes, highlighting existing disparities, and identifying strategies for supporting pregnant capable individuals to actualize their reproductive goals.

Community-level interventions are indicated, including comprehensive school-based sexual health education, group-level prenatal care, and breastfeeding support groups. SIPI may also be mitigated through enhanced access to patient-centered contraceptive counseling and the full range of contraceptive methods (Teal, 2014). Policy-level implications indicate a need to enhance access to perinatal healthcare in rural areas, including local clinics and birthing facilities, to mitigate the closure of labor and delivery units (Radke et al., 2023). Additionally, policies to protect Certified Nurse Midwives to work at the fullest extent of their training and licensure are important for expanding perinatal health care services (Carlough et al., 2021).

Building on existing frameworks for social capital and subjective norms, future research with robust measures will deepen our understanding of the influence of neighborhood cohesion, social networks, and peer influence on sIPI. Specifically, qualitative methods are indicated to explore women's and families' perceptions of the impact of social influences and lack of access to health care services on key decisions regarding family planning and birth spacing. Additionally, future research should leverage GIS and spatial analyses to investigate geographic disparities in sIPI and examine spatial autocorrelation in social and structural factors—such as healthcare access and socioeconomic conditions—across geographically diverse communities.

## CRediT authorship contribution statement

**Qian Huang:** Writing – review & editing, Writing – original draft, Visualization, Validation, Methodology, Formal analysis, Data curation, Conceptualization. **Liane M. Ventura:** Writing – review & editing, Writing – original draft, Conceptualization. **Jo Bradley:** Writing – review & editing. **Kate Beatty:** Writing – review & editing, Conceptualization.

## Funding sources

This work was supported by the Dogwood Health Trust (grant #2747).

## Declaration of competing interest

The authors declare that they have no known competing financial interests or personal relationships that could have appeared to influence the work reported in this paper.

## Figures and Tables

**Appendix 1:** Ordinary least squares regression results for short interpregnancy interval and social and behavioral predictors, North Carolina, 2018–2022.

	Coefficient	Std. Error	t	Probability	VIF
(Constant)	13.4	4.1	3.23	0.0	–
Race	0.05	0.03	1.73	0.1	4.89
Maternal Age	0.13	0.05	2.50	0.0	1.53
Education	−0.08	0.04	−1.94	0.1	2.53
Poverty	−0.06	0.08	−0.71	0.5	2.27
Household Vehicle Access	0.28	0.14	1.92	0.1	2.83
Smoking during Pregnancy	0.01	0.09	0.12	0.9	3.27
Prenatal Care	−0.04	0.04	−1.04	0.3	1.34
Breastfeeding at Discharge	−0.05	0.04	−1.19	0.2	4.50

**Appendix 2:** Spatial lag model results for short interpregnancy interval in North Carolina, 2018–2022.

	Coefficient	Std. Error	z-value	Probability
W_sIPI	0.58	0.09	6.28	<0.001
(Constant)	5.35	3.48	1.54	0.1
Race	0.03	0.02	1.53	0.1
Maternal Age	0.11	0.04	2.49	0.0
Education	−0.04	0.03	−1.11	0.3
Poverty	−0.07	0.07	−1.08	0.3
Household Vehicle Access	0.21	0.12	1.80	0.1
Smoking during Pregnancy	0.05	0.07	0.62	0.5
Prenatal Care	−0.02	0.03	−0.68	0.5
Breastfeeding at Discharge	−0.05	0.04	−1.51	0.1

## Data Availability

Data will be made available on request.

## Appendix B Supplementary data

Supplementary material Image 1
