# Supplementary material for: Geospatial analysis of area-level social and behavioral factors associated with short interpregnancy intervals in North Carolina, U.S
Source: Prev Med Rep. 2025 Aug 22;58:103216. doi: 10.1016/j.pmedr.2025.103216 (PMC12398778; doi:10.1016/j.pmedr.2025.103216)
Supplement: Supplementary file 1 — Supplementary material [file mmc1.pdf]

EAST TENNESSEE STATE UNIVERSITY

Human Research Protection Program

PO Box 70565
Johnson City, TN 37614
P (423) 439-6053
E hrpp@etsu.edu

Full AAHRPP Accreditation
Association for the Accreditation of Human Research Protection Programs, Inc.

RE: Determination of Not Human Subjects Research

Submitter:	Qian Huang
Department:	Biostatistics & Epidemiology
Project Title:	Geospatial analysis of area-level social and behavioral factors associated with short interpregnancy intervals in North Carolina, US
Submission Type:	IRB Form 129 (Research Determination Request)
Determination Date:	June 16, 2025

Thank you for submitting information about your project referenced above. It was determined that this proposed activity does not meet the definition of research involving human subjects. Therefore, it does not fall under the purview of the ETSU IRB.

East Tennessee State University IRB review and approval is not required. This determination applies only to the activities described in the submission and does not apply should any changes be made. If changes are made and there are questions about whether the activities are human subjects research in which the organization is engaged, please submit a new request for a determination.

Please note that your project may be subject to other rules or oversight. For example, if your project is subject to the HIPAA Privacy Rule or General Data Protections Regulations (GDPR), you are responsible for consulting with ETSU Legal (if subject to ETSU) to ensure that your project is compliant with applicable regulations.

Please note that if this project is subject to Department of Defense (DoD) oversight, DoD must concur with this determination before this activity can begin. You are responsible for obtaining concurrence as appropriate.

For any assistance, please contact the Human Research Protection Program at HRPP@etsu.edu or (423)-439-6053.
